# Resilience: A Protective Factor from Depression and Anxiety in Mexican Dialysis Patients

**DOI:** 10.3390/ijerph182211957

**Published:** 2021-11-14

**Authors:** Cristina J. González-Flores, Guillermo García-García, Abel Lerma, Héctor Pérez-Grovas, Rosa M. Meda-Lara, Rebeca M. E. Guzmán-Saldaña, Claudia Lerma

**Affiliations:** 1 Centro Universitario de la Cienega, University of Guadalajara, Ocotlán 47820, Mexico; cristina.gonzalez4243@academicos.udg.mx; 2 Nephrology Department, Civil Hospital de Guadalajara Fray Antonio Alcalde, Guadalajara 44280, Mexico; ggarcia1952@gmail.com; 3 Institute of Health Sciences, Universidad Autónoma del Estado de Hidalgo, San Juan Tilcuautla 42160, Mexico; abel_lerma@uaeh.edu.mx (A.L.); rguzman@uaeh.edu.mx (R.M.E.G.-S.); 4 National Institute of Cardiology Ignacio Chávez, México City 14080, Mexico; hpgrovas@gmail.com; 5 Departments of Basic Psychology and Medical Clinics, Health Sciences Center, University of Guadalajara, Guadalajara 44340, Mexico; rosammeda2013@gmail.com

**Keywords:** anxiety, cognitive distortions, depression, dialysis, ESRD, quality of life, resilience

## Abstract

Depression and anxiety are highly prevalent psychological disorders in end-stage renal disease (ESRD) that have a negative clinical impact. The purpose of our study was to identify factors associated with the presence of depression and anxiety, in a sample of ESRD patients treated with hemodialysis. We included 187 patients from two dialysis facilities, age 18–65 years. Beck’s depression and anxiety inventories, KDQOL36 questionnaire, the cognitive distortion scale and the Mexican scale of resilience were used. Socio-demographic and clinical information was obtained from medical records. Depression was present in 143 (76.4%) patients. Patient with depression were older (33 (26–52) years vs. 30 (24.43) years, *p* = 0.025), had a lower education level (36% vs. 9%, *p* = 0.001), used more medications (67% vs. 36%, *p* = 0.001), had a comorbidity (75% vs. 41%, *p* = 0.001), and a higher proportion were waiting for a kidney transplant. Anxiety was present in 112 (59.8%) cases. By multivariate analysis, depression was independently associated with lower education, absence of previous kidney transplant, anxiety, higher cognitive distortion, lower psychological resilience, and lower quality of life scores. In conclusion, lower psychological resilience, lower education level, and higher cognitive distortions are factors associated with depression and anxiety in ESRD patients.

## 1. Introduction

Advances in the treatment of end-stage renal disease (ESRD) have improved the quality of life and survival of patients receiving renal replacement therapy (RRT) [1]. However, most actions have focused on clinical outcomes with disregard of the psychosocial aspects of ESRD [2]. Depression and anxiety are the most prevalent psychological disorders in ESRD patients, and they increase the risk of negative outcomes such as frequent hospitalization, poor quality of life, and a higher risk of death [3,4].

The relationship between the presence of depression, anxiety, and other psychological and socio-demographic factors in ESRD patients varies between studies [5,6]. Social support, family issues, dialysis unit culture, and socio-economic status are among the factors linked to depression and anxiety in these patients [7]. Depression is the consequence of the interpretation of unfortunate events from errors of thought represented schematically. The perception of the disease through negative emotional states, such as depression and anxiety, are expressed through automatic thoughts, and this information process can be objective or distorted, which leads to thinking errors defined as cognitive distortions [8,9]. It has been proposed that depression and anxiety symptoms are often mediated by distorted thoughts which lead to a negative emotional state [7,10]. Additional psychological factors associated with depression in these patients are cognitive appraisal and coping process [11]. From a positive salutogenic approach it is possible to change the negative perception of the disease [12]. Resilience is a construct that has been defined from a broad range of models and, because there is not a shared definition, the concept of resilience is complex. However, the resilience is much more than resistance to trauma—it expresses the ability to react positively despite difficulties, turning them into opportunities for growth [13]. Resilience consists of personalized skills to cope to adversity situations and even emerge stronger from them. In chronic disease, resilience can be associated with adherence to treatment and well-being [10,11,14,15,16,17,18,19]. Although there is evidence that psychological resilience acts as a protective factor against depression and anxiety, resilience has been scarcely evaluated in ESRD patients [15,16,18,19,20,21,22,23,24]. The dimensions of resilience (i.e., strength, self-confidence, social competence, social support, family support and self-structure) can be mediators in the reduction in negative emotional states in ESRD patients [14].

Chronic kidney disease (CKD) is a public health problem in Mexico. Between 1990 to 2017, the age-standardized mortality rate due to CKD increased from 28.7 to 58.1 deaths per 100,000 population (a 102% rise), while the years of life lost and disability-adjusted life-years increased 116% and 94%, respectively [25]. By 2016, the treated ESRD incidence and prevalence rates were 355 and 1477 per million population, respectively [26]. However, there is a paucity of studies evaluating depression and anxiety in Mexican patients on renal replacement therapy (RRT). Symptoms of depression have been reported in 25% to 56% of Mexican adult patients on dialysis [7,27,28,29]. Similarly, moderate to severe symptoms of depression have been found in Mexican children and adolescents on RRT [30]. The study of resilience in the renal population has shown that the construct is present in various dimensions in the life of the patient and has a mediating effect in relation to other psychological and contextual variables [21,23].

The purpose of our study was to explore potential factors associated with the presence of depression and anxiety in a sample of ESRD patients treated with hemodialysis. We evaluated socio-demographic and clinical characteristics as well as quality of life, resilience and cognitive distortions.

## 2. Materials and Methods

### 2.1. Patients

The study included a non-probabilistic sample of 187 ESRD patients, treated with dialysis at the Hospital Civil de Guadalajara Fray Antonio Alcalde, Jalisco, and at the Instituto Nacional de Cardiología, Mexico City, two large tertiary-health care facilities for dialysis patients without health care insurance. Patients >18 years of age who signed the informed consent were included. The study was approved by the Committee of Research and Bioethics our institution (protocol number CB/023/2017). The study included only participants with complete collection of data for all variables.

The study sample included 187 patients, with a median age of 32 (25–48) years and median vintage time of 75 (15–63) months. Seventy-nine patients (42.2%) were female, 55 (29.4%) had completed only primary school education, 176 (94.1%) lived with a companion, 57 (30.4%) were employed, 154 (82.3%) had no economic dependents, 112 (59.8%) used medications, 125 (66.8%) had other comorbidities, 75 (40.1%) were awaiting a kidney transplant, 11 (5.8%) were smokers, and 60 (32.0%) were past smokers. The number of patients with comorbidities were: arterial hypertension 89 (47.6%), diabetes mellitus 18 (9.6%), lupus 5 (2.7%), ischemic cardiopathy 3 (1.6%), hypertrophic cardiopathy 4 (2.1%), hypothyroidism 4 (2.1%), and other non-specified 2 (1.1%).

### 2.2. Data Collection

A member of our research team (C.J.G-F) invited each patient who fulfilled the inclusion criteria, explained the nature of the study, and answered any question from the patients. Those who accepted to participate signed an informed consent form. Then, they answered the self-report questionnaires. The application was carried out during the hemodialysis shifts of the clinics in the waiting room before entering the hemodialysis sessions. In each participant, the following variables were collected directly from the patient in a data sheet: age, sex, education level, employment status, living arrangements (living alone or with a companion), number of dependents, medication use, comorbidities, previous kidney transplant, awaiting kidney transplantation, smoking habits, and dialysis vintage. Blood glucose, hemoglobin, serum albumin, and serum creatinine were obtained from the most recent evaluation annotated in the clinical records (within one month of enrollment).

Depression was assessed with the Spanish version of the Beck Depression Inventory (BDI) [31], which has been validated in the Mexican population. The BDI has 21 items, and the total score is obtained by adding the values of the selected sentences, which have a Likert’s scale range of 0–3. The range of the score obtained is 0–63 points. The questionnaire has two dimensions: cognitive symptoms and somatic symptoms. The BDI has a reliability measure (Cronbach’s alpha) of 0.87 [7]. Anxiety was assessed with the Spanish version of the Beck Anxiety Inventory (BAI) [32], also validated in the Mexican population. The BAI has 21 items to assess somatic symptoms and cognitive symptoms; each item has a Likert’s scale range of 0–3 and a total score range of 0–63 points. The BAI has a Cronbach’s alpha of 0.90. Both BDI and BAI have been widely used in the general population and in ESRD patients [7,33,34,35].

Quality of life was evaluated with the KDQOL 36 (Kidney Disease Quality of Life) questionnaire, developed by the Renal Disease Quality of Life Working Group with the intention of having a self-report measure that assesses the quality of life in patients with ESRD. The short version KDQOL 36 (1.3) was used in this study, which has 36 items (12 items are questions about perception of quality of life and 24 items are directly related to ESRD). The questionnaire is divided into five dimensions: (1) Physical component, (2) Mental component, (3) Burden of disease, (4) Symptoms and problems, and (5) Effects of kidney disease. The total score ranges from 0 to 100. The KDQOL 36 was validated in the Mexican population and has a Cronbach´s alpha of 0.80 [36].

Psychological resiliency was evaluated with the Mexican scale of resilience, which has 43 items grouped in 5 factors (strength and self-confidence, social competence, family support, social support, and structure), and has a Cronbach´s alpha of 0.93. The resilience inventory was designed for the Mexican population, and subsequently validated in ESRD patients specifically with a Cronbach’s alpha of 0.96 (the alpha varies in the five factors from 0.85 to 0.95) [14].

Distorted thoughts were evaluated with a questionnaire developed and validated previously for ESRD patients, which includes 30 items with a Likert’s scale ranging from 1 to 5 [9]. The distorted thoughts scale comprises four factors (perfectionism, catastrophism, negative self-labelling, and dichotomous thinking) and has a Cronbach´s alpha of 0.93 [9].

### 2.3. Statistical Analysis

Statistical analysis was performed using the computer program SPSS version 21.0 (IBM Corp, Armonk, NY, USA). Categorical variables are described as absolute value and percentage and compared between groups by χ^2^ tests. Most continuous variables were not normally distributed (Smirnov–Kolmogorov test, *p* < 0.05); these are described with medians (percentile 25–percentile 75) and were compared between groups by Mann–Whitney U tests. A Spearman correlation analysis between total scores of psychological variables and quality of life was performed.

Each variable that had a significant difference between the groups with or without depression symptoms, the association with the presence of symptoms of depression (total depression score ≥ 10 points) by binary logistic regression analysis, and results are reported as odds ratio (95% confidence interval). The odds ratio of each variable was estimated with a univariate binary logistic regression model. The presence of depression was considered a dependent variable, and univariate binary logistic regression models were calculated with each of the following independent variables: age, female sex, primary school, drug use, comorbidities, previous transplant, waiting transplant, anxiety score, resilience score, and distorted thoughts score. Then, multivariate logistic regression models were used to calculate adjusted odds ratios for each variable. A base regression model included all the socio-demographic variables associated with the presence of depression (age, female sex, primary school, drug use, comorbidities, previous transplant, waiting transplant). Then, other multivariate logistic regression models were used to estimate the adjusted odds ratios for psychological variables and quality of life, by considering all socio-demographic variables of the base model and adding one psychological variable in each model. This allows the estimation of the magnitude of the association between each psychological variable and quality of life with the presence of depression, by adjusting for the socio-demographic variables that could be considered confounding factors. Similarly, binary logistic regression models were constructed to explore the association between the presence of anxiety (total anxiety score ≥ 7 points) as the dependent variable and the variables that had significant differences between groups with or without the presence of anxiety (age, female sex, albumin, working status, previous transplant, depression score, resilience score, and distorted thoughts score) as independent variables. Univariate binary regression logistic models were tested for each independent variable and then a base multivariate model was tested with the combination of albumin, working status, and previous transplant (as independent variables).

Then, other regression models were built with the base model and each of the psychological variables. This allowed the estimation of the magnitude of the association between each psychological variable and quality of life with the presence of anxiety, by adjusting for the socio-demographic variables that could be considered confounding factors. A detailed description of the regression models is in the Supplementary Material (Section S1).

## 3. Results

Symptoms of depression (BDI ≥ 10 points) were present in 143 (76.4%) cases (Table 1). Patients with depressive symptoms were older (33 (26–52) years vs. 30 (24.43) years, *p* = 0.025), had a lower education level, i.e., primary school (36% vs. 9%, *p* = 0.001), used more medications (67% vs. 36%, *p* = 0.001), had more comorbidities (75% vs. 41%, *p* = 0.001), and were less likely to be waiting for a kidney transplant, in comparison to patients without depression. Additionally, patients with depression had higher anxiety and cognitive distortions scores, in addition to a lower psychological resilience and a lower perception of quality of life).

Anxiety (total anxiety score ≥ 7 points) was present in 112 (59.8%) cases (Table 2). Patients with anxiety were less likely to be employed and to have had a previous kidney transplant and had lower serum albumin. Additionally, they had a higher depression score, lower psychological resilience, lower quality of life scores, and a higher cognitive distortion score. There were no significant differences between groups in all other variables.

According to the bivariate correlation analysis (Table 3), variables belonging to negative emotional states such as depression, anxiety, and cognitive distortions maintained a direct correlation between their scores and an inverse correlation with resilience and quality of life scores, whereas the latter were positively correlated with each other.

The univariate regression analysis (Table 4) showed that all the socio-demographic, psychological, and quality of life variables included in the univariate models were associated with the presence of depression symptoms. However, in the multivariate regression analysis, depression was independently associated with lower education, previous kidney transplant, anxiety, higher cognitive distortion, lower psychological resilience, and lower perception of quality of life (Table 4).

The results of logistic regression analysis to assess the variables associated with the presence of anxiety are shown in Table 5. Multivariate analysis showed that depression, higher cognitive distortions, and lower quality of life scores were independently associated with the presence of anxiety symptoms.

## 4. Discussion

Depression and anxiety were highly prevalent in Mexican ESRD patients treated by hemodialysis. Older age, lower education, absence of a previous transplant, anxiety, higher cognitive distortions, lower psychological resilience, and lower perception of quality of life were independently associated with the presence of depression. Furthermore, the presence of anxiety symptoms was independently related to the scores of depression score, cognitive distortions, and lower perception of quality of life. The prevalence of depression in ESRD patients has been found to range from 10% to 70% depending on the evaluation tools and cut-off values applied to define the presence of depression [6,37]. In our population, the presence of depressive symptoms (76%) and anxiety symptoms (60%) was similar to other reports [38,39], but higher than previously reported in ESRD Mexican patients on dialysis [27].

In the quest to understand the factors related to depression and anxiety in ESRD patients, many previous studies from different populations have been undertaken, with widely varying findings, as shown with relevant examples in Table S1 (Supplementary Material). Even age and female sex, which have been considered factors highly related to depression and anxiety [4,5] are variables not always associated with these psychopathologies [18,40]. The sample of ESRD patients studied in the present work showed no independent association of depression or anxiety with the patient’s age and gender. Moreover, similar to a previous report [41], in our study, having a lower education level (i.e., primary school) was associated with the presence of depression symptoms compared to patients who finished secondary school or a higher level. However, other studies have not found similar results [5,42]. Additionally, having a previous transplant was associated with lower probability of depression. [40,43] We speculate that having a previous trans-plant may increase the patient’s perception of better life expectancy because it entails an improvement in the quality of life without the invasive substitute treatments [44]. However, testing such a causal association requires further studies.

Lower albumin levels have been previously associated with anxiety symptoms [45,46]. However, albumin was not an independent factor for the presence of anxiety in our population, and other studies have actually reported that high albumin levels were associated with physical states of agitation or anxiety [47,48]. Low levels of albumin are related to malnutrition and both have been associated with anxiety [45,46]. Because malnutrition and other comorbidities of renal disease may increase somatic symptoms, it is important to consider biochemical and physiological markers as possible confounding variables in the research of psychological pathologies in ESRD patients.

Regarding other psychological variables, cognitive distortions, psychological resilience, and quality of life were independently associated with depression, whereas anxiety was also related to those variables except resilience. Misperception and cognitive distortion in ESRD patients have been previously documented [9]. Furthermore, several studies have shown that low resilience was associated with the presence of physical and psychological disorders [15,49,50]. Therefore, psychological resilience is considered a protective factor of both physical and mental health [11] and is associated with a better quality of life [51].

The present study is the first report evaluating the association between resiliency and the presence of anxiety and depression symptoms in ESRD Mexican patients on dialysis. Our findings suggest that it would be useful to implement comprehensive programs for better patient understanding of ESRD disease and to decrease psychological pathologies. Psychological interventions with evidence of results for the reduction in pathologies have become a necessity for the comprehensive treatment of medically ill populations. The promotion of salutogenic factors can serve as an alternative to improve the emotional well-being of the patient. Resilience can be considered as a mediator or moderator concept in reducing pathologies [14,52]. Psycho-educational tools have constituted an effective proposal for reducing gaps caused by a low educational level [53], because they allow a greater understanding of the characteristics of the disease and improve adherence to treatment [54,55]. Our results also suggest the need for psychological interventions aimed at reducing symptoms of depression and anxiety in the dialysis population, to improve dimensions of quality of life in the patient. The effectiveness of psychological programs aimed at reducing symptoms of depression and anxiety has been proven [7,56,57]. Nevertheless, other psychological interventions that include salutogenic variables such as resilience may enhance the effectiveness of such interventions [58].

Some previous studies have previously associated resilience to variable negative emotional states such as depression, anxiety, and stress [18,59,60,61,62,63,64], and to positive emotional states such as well-being and social support [65,66]. This is a construct that has been present in a chronically ill population, including hemodialysis patients [20,67,68]. The present study agrees with the literature, finding associations of resilience with depression and anxiety in ESRD patients, which suggests that resilience may function as a protective factor against these symptoms. Resilience acts as a personality factor and promotes physical and mental health. The study and the immersion of the construct in other disciplines are of interest in various areas such as the social and health sciences [14,69]. In health settings, the concept of resilience is widely used and understood as the capacity for adaptive response to the disease as a situation of adversity [69]. The epidemiological transition has led to chronic degenerative diseases, which are commonly accompanied by negative emotional states such as depression and anxiety symptoms, resulting in the health system focusing on such conditions [65]. The psychological impact and overload of the disease justify greater attention to the patient´s positive coping mechanisms because resilience is a vital element in psychological treatment for this population. Our study also negatively associated distorted thoughts with resilience; in previous studies using another conceptual framework, variables such as cognitive rumination were associated with protective variables such as post-traumatic growth [16].

### 4.1. Study Limitations

A limitation of our study is that it is based on a relatively small non-randomly selected sample of dialysis patients who had no health insurance from two geographical areas of Mexico. Patients without health care insurance in Mexico (self-employed, unemployed, workers in the informal sector, peasants) usually belong to the country’s lower socio-economic status, which is a risk factor for depression [70] and poorer clinical outcomes. However, a broader sample from patients from other geographical regions of Mexico and other socio-economic status is needed to increase the external validity of the present results. Further longitudinal studies are required to address the influence of psychological resilience and distorted thoughts on relevant clinical outcomes such as hospitalizations and mortality. The observational transverse study design of the present study allowed the exploration of potential variables associated with the presence of depression or anxiety. Future work with study designs that consider a priori hypotheses are required to identify causal associations with depression and anxiety.

### 4.2. Implications for Practice

Psychological interventions with evidence of results for the reduction in pathologies have become a necessity for the comprehensive treatment of medically ill populations. The promotion of salutogenic factors can serve as an alternative to improve the emotional well-being of the patient. Resilience can be considered as a mediator or moderator concept in reducing pathologies [52]. Results of this study may aid in preparing appropriate psychosocial interventions in which resiliency plays a core role, which could positively impact the psychological sphere of patients living with ESRD. For example, using resilience skills, patients may recover strength and self-confidence, increase social competence, social support, family support, and self-structure. This would have a subsequent positive impact in their activities, life, routines, goals, and time [14].

## 5. Conclusions

Depression and anxiety are highly prevalent in ESRD Mexican patients treated by hemodialysis. Low education level, decreased psychological resilience, and increased cognitive distortions are factors associated with depression and anxiety. Interventions aimed to reduce depression and anxiety in this population may generate behavioral changes and impact the quality of life of these patients.

## Figures and Tables

**Table 1:** Socio-demographic, clinical characteristics, and psychological variables of patients with and without depression (total BDI score ≥ 10 points). Data is shown as median (percentile 25–percentile 75) or absolute value (percentage).

Variables	Presence of Depression		*p*-Value
	Yes (*n* = 143)	No (*n* = 44)	
Age (years)	33 (26–52)	30 (24–43)	0.03
Female (%)	59 (41%)	20 (45%)	0.62
Schooling (%)			
Primary school	51 (36%)	4 (9%)	<0.01
Secondary school or higher	92 (64%)	40 (91%)	
Living status (%)			
Alone	6 (4%)	5 (11%)	0.08
With companion	137 (96%)	39 (89%)	
Employed (%)	40 (28%)	17 (39%)	0.18
With dependents (%)	27 (18%)	6 (14%)	0.43
On medications (%)	96 (67%)	16 (36%)	<0.01
With other comorbidities (%)	107 (75%)	18 (41%)	<0.01
Previous kidney transplant (%)	10 (7%)	14 (32%)	<0.01
Awaiting kidney transplantation (%)	49 (34%)	26 (59%)	<0.01
Smoking (%)	9 (6%)	2 (4%)	0.66
Past smoker (%)	48 (33%)	12 (27%)	0.43
Dialysis vintage (months)	39 (18–60)	36 (12–67)	0.40
Serum creatinine (mg/dL)	7.9 (6.9–8.7)	8.1 (6.4–8.8)	0.98
Blood Hemoglobin (mg/dL)	8.9 (8.1–9.7)	9.1 (8.2–9.9)	0.64
Serum albumin (g/dL)	3.6 (3.3–3.9)	3.8 (3.4–4.1)	0.18
Serum glucose (mg/dL)	97 (87–106)	95 (88–102)	0.67
Anxiety score	11 (7–17)	5 (3–9)	<0.01
Resiliency score	122 (117–130)	130 (125–153)	<0.01
Quality of life score	65 (54–70)	73 (64–83)	<0.01
Cognitive distortion score	63 (50–80)	44 (40–55)	<0.01

**Table 2:** Socio-demographic, clinical characteristics, and psychological variables of patients with and without anxiety (total BAI score ≥ 10 points). Data is shown as median (percentile 25–percentile 75) or absolute value (percentage).

Variables	Presence of Anxiety		*p*-Value
	Yes (*n* = 112)	No (*n* = 75)	
Age (years)	33 (25–52)	33 (26–52)	0.66
Female (%)	46 (41%)	33 (44%)	0.69
Schooling (%)			0.32
Primary school	36 (32%)	19 (25%)	
Secondary school or higher	76 (68%)	56 (75%)	
Living status (%)			0.31
Alone	5 (5%)	6 (8%)	
With companion	107 (95%)	69 (92%)	
Employed (%)	28 (25%)	29 (39%)	0.05
With dependents (%)	19 (17%)	14 (19%)	0.51
On medications (%)	41 (55%)	96 (58%)	0.23
With other comorbidities (%)	79 (70%)	46 (61%)	0.19
Previous kidney transplant (%)	10 (9%)	14 (19%)	0.05
Awaiting kidney transplantation (%)	42 (37%)	33 (44%)	0.37
Smoking (%)	6 (5%)	5 (7%)	0.71
Past smoker (%)	32 (29%)	28 (37%)	0.21
Dialysis vintage (months)	36 (12–57)	42 (24–72)	0.08
Serum creatinine (mg/dL)	8.1 (7.2–8.7)	7.8 (6.4–8.6)	0.27
Blood Hemoglobin (mg/dL)	8.9 (8.2–9.6)	8.9 (8.2–10.1)	0.35
Serum albumin (g/dL)	3.6 (3.3–3.8)	3.8 (3.4–4.1)	0.03
Serum glucose (mg/dL)	97 (85–106)	97 (88–106)	0.78
Depression score	18 (13–25)	11 (6–15)	<0.01
Resiliency score	123 (117–130)	127 (119–145)	0.02
Quality of life score	64 (53–71)	70 (61–81)	<0.01
Cognitive distortion score	66 (48–83)	51 (40–73)	<0.01

**Table 3:** Spearman correlation analysis between psychological variables. The correlation coefficients were calculated from the total score of each variable.

	Depression	Anxiety	Resiliency	Cognitive Distortions
Anxiety	0.599 **			
Resilience	−0.353 **	−0.238 **		
Cognitive distortions	0.472 **	0.445 **	−0.354 **	
Quality of life	−0.431 **	−0.337 **	0.274 **	−0.320 **

** *p*-value < 0.01.

**Table 4:** Binary logistic regression analysis to assess the variables associated with the presence of depression (dependent variable). Results are shown as odds ratio (C.I.95%).

Variables	Univariate Models	Multivariate Models
Age (years)	1.027 (1.003–1.051)	0.995 (0.967–1.024) ^1^
Female sex *	0.843 (0.427–1.664)	0.644 (0.286–1.449) ^1^
Primary education ^&^	5.543 (1.876–16.377)	5.479 (1.601–18.752) ^1^
Use any medications †	3.574 (1.764–7.244)	1.982 (0.747–5.259) ^1^
Any comorbidities ‡	4.293 (2.111–8.730)	1.867 (0.703–4.961) ^1^
Previous transplant ¶	0.161 (0.065–0.397)	0.178 (0.063–0.505) ^1^
Waiting transplant ◊	0.361 (0.180–0.722)	0.416 (0.180–0.957) ^1^
Anxiety score (points)	1.261 (1.144–1.390)	1.237 (1.110–1.380) ^2^
Resiliency score (points)	0.964 (0.945–0.984)	0.975 (0.954–0.996) ^3^
Cognitive distortions score (points)	1.056 (1.030–1.082)	1.046 (1.019–1.074) ^4^
Quality of life score (points)	0.947 (0.920–0.975)	0.953 (0.922–0.984) ^5^

* Reference value was male sex; ^&^ Reference value was secondary school or higher; † Reference value was not using any medication; ‡ Reference value was not having any comorbidity; ¶ Reference value was not having a previous transplant; ◊ Reference value was not waiting for a transplant. ^1^ The base model included the following variables: age, female sex, primary education, use of any medications, any comorbidity, previous transplant, waiting transplant. ^2^ Includes the base model + anxiety score. ^3^ Includes the base model + resiliency score. ^4^ Includes the base model + cognitive distortions score. ^5^ Includes the base model + quality of life score.

**Table 5:** Binary logistic regression analysis to assess the variables associated with the presence of anxiety (dependent variable). Results are shown as odds ratio (C.I.95%).

Variables	Univariate Models	Multivariate Models
Age (years)	0.998 (0.981–1.016)	0.994 (0.975–1.012) ^1^
Female sex *	0.887 (0.491–1.602)	0.726 (0.380–1.385) ^1^
Albumin (g/dL)	0.554 (0.294–1.044)	0.609 (0.315–1.178) ^1^
Currently working ^&^	0.529 (0.281–0.994)	0.509 (0.253–1.023) ^1^
Previous kidney transplant †	0.427 (0.179–1.021)	0.573 (0.224–1.462) ^1^
Depression score (points)	1.143 (1.088–1.201)	1.140 (1.083–1.201) ^2^
Resiliency score (points)	0.982 (0.966–0.999)	0.984 (0.967–1.000) ^3^
Cognitive distortions score (points)	1.035 (1.018–1.053)	1.032 (1.015–1.050) ^4^
Quality of life score (points)	0.959 (0.937–0.981)	0.961 (0.938–0.985) ^5^

* Reference value was male sex; ^&^ Reference value was not having a current work; † Reference value was not having a previous transplant. ^1^ The base model included the following variables: albumin (g/dL), currently working, previous kidney transplant. ^2^ Included the base model + depression score. ^3^ Included the base model + resiliency score. ^4^ Included the base model + cognitive distortions score. ^5^ Included the base model + quality of life score.

## Data Availability

The data presented in this study are available on request from the corresponding author.

## Supplementary Materials

The following are available online at https://www.mdpi.com/article/10.3390/ijerph182211957/s1, Table S1: Relevant previous research regarding the factors associated with depression and anxiety, distorted thoughts, and psychological resilience in ESRD patients treated with hemodialysis.; Section S1: Detailed description of the logistic regression analysis.

## References

[1] Cusumano A.M., Bedat M.C.G. (2008). Chronic Kidney Disease in Latin America: Time to Improve Screening and Detection. Clin. J. Am. Soc. Nephrol..

[2] Delgado-Domínguez C.J., Sanz-Gómez S., López-Herradón A., Díaz Espejo B., Lamas González O., de los Santos Roig M., Berdud Godoy I., Rincón Bello A., Ramos Sánchez R. (2021). Influence of Depression and Anxiety on Hemodialysis Patients: The Value of Multidisciplinary Care. Int. J. Environ. Res. Public Health.

[3] Lopes A.A., Bragg J., Young E., Goodkin D., Mapes D., Combe C., Piera L., Held P., Gillespie B., Port F.K. (2002). Depression as a Predictor of Mortality and Hospitalization among Hemodialysis Patients in the United States and Europe. Kidney Int..

[4] Kellerman Q.D., Christensen A.J., Baldwin A.S., Lawton W.J. (2010). Association between Depressive Symptoms and Mortality Risk in Chronic Kidney Disease. Health Psychol..

[5] Nisar S., Uzair A., Khan M.A., Akhtar S. (2017). Association of Depression with Socio-Demographic Factors in Patients Undergoing Henodialysis. Depress. Hemodial. Pak. Armed Forces Med. J..

[6] Kimmel P., Peterson R. (2008). Depression in End-Stage Renal Disease Patients Treated With Hemodialysis: Tools, Correlates, Outcomes, and Needs. Semin. Dial..

[7] Lerma A., Perez-Grovas H., Bermudez L., Peralta-Pedrero M.L., Robles-García R., Lerma C. (2017). Brief Cognitive Behavioural Intervention for Depression and Anxiety Symptoms Improves Quality of Life in Chronic Haemodialysis Patients. Psychol. Psychother. Theory Res. Pract..

[8] Rnic K., Dozois D.J.A., Martin R.A. (2016). Cognitive Distortions, Humor Styles, and Depression. Eur. J. Psychol..

[9] Lerma A., Salazar E., Perez-Grovas H., Bermudez L., Gutiérrez D., Reyes I., Bochicchio T., Robles-García R., Lerma C. (2012). Desarrollo y Validación de Un Instrumento Para La Evaluación de Distorsiones Cognitivas En Pacientes Con Insuficiencia Renal Crónica Terminal. Salud Ment..

[10] Wyssen A., Debbeler L.J., Meyer A.H., Coelho J.S., Humbel N., Schuck K., Lennertz J., Messerli-Bürgy N., Trier S.N., Isenschmid B. (2018). Relevance of the Thought-Shape Fusion Trait Questionnaire for Healthy Women and Women Presenting Symptoms of Eating Disorders and Mixed Mental Disorders. Clin. Psychol. Psychother..

[11] Chan R., Steel Z., Brooks R., Heung T., Erlich J., Chow J., Suranyi M. (2011). Psychosocial Risk and Protective Factors for Depression in the Dialysis Population: A Systematic Review and Meta-Regression Analysis. J. Psychosom. Res..

[12] Bauer G.F., Roy M., Bakibinga P., Contu P., Downe S., Eriksson M., Espnes G.A., Jensen B.B., Juvinya Canal D., Lindström B. (2020). Future Directions for the Concept of Salutogenesis: A Position Article. Health Promot. Int..

[13] Sisto A., Vicinanza F., Campanozzi L.L., Ricci G., Tartaglini D., Tambone V. (2019). Towards a Transversal Definition of Psychological Resilience: A Literature Review. Medicina.

[14] Lerma A., Ordónez G., Mendoza L., Salazar-Robles E., Rivero J., Pérez-Granados E., Pérez-Grovas H., Ruiz-Palacios P., Ibarra A., Lerma C. (2019). Psychometric Properties of the Resilience Scale in Mexican Patients with Chronic Hemodialysis. Salud Ment..

[15] Freire C.M.M., Arantes E.P., de Tajra R.D.P., Santiago H.R., Carvalho A.F., Libório A.B. (2017). Resilience, Religiosity and Treatment Adherence in Hemodialysis Patients: A Prospective Study. Psychol. Health Med..

[16] Li T., Liu T., Han J., Zhang M., Li Z., Zhu Q., Wang A. (2018). The Relationship among Resilience, Rumination and Posttraumatic Growth in Hemodialysis Patients in North China. Psychol. Health Med..

[17] Kim G.M., Lim J.Y., Kim E.J., Park S. (2019). Resilience of Patients with Chronic Diseases: A Systematic Review. Health Soc. Care Community.

[18] Moreira J.M., Bouissou Morais Soares C.M., Teixeira A.L., Simões e Silva A.C., Kummer A.M. (2015). Anxiety, Depression, Resilience and Quality of Life in Children and Adolescents with Pre-Dialysis Chronic Kidney Disease. Pediatric Nephrol..

[19] Ma L.-C.C., Chang H.-J.J., Liu Y.-M.M., Hsieh H.-L.L., Lo L., Lin M.-Y.Y., Lu K.-C.C. (2013). The Relationship between Health-Promoting Behaviors and Resilience in Patients with Chronic Kidney Disease. Sci. World J..

[20] Kim E.Y., Lee Y.-N., Chang S.O. (2020). Measuring the Resilience of Patients on Hemodialysis: Development of a Patient on Hemodialysis Resilience Scale. Nephrol. Nurs. J..

[21] Kim E.Y., Lee Y.-N., Chang S.O. (2019). How Do Patients on Hemodialysis Perceive and Overcome Hemodialysis?: Concept Development of the Resilience of Patients on Hemodialysis. Nephrol. Nurs. J..

[22] Shakoor E., Jahromi M.K., Salesi M., Sadeghi H. (2015). The Effects of 10 Weeks Concurrent Aerobic and Strength Exercise on Quality of Life and Resilience of Kidney Transplant Patients. Int. J. Appl. Exerc. Physiol..

[23] Kukihara H., Yamawaki N., Ando M., Nishio M., Kimura H., Tamura Y. (2020). The Mediating Effect of Resilience between Family Functioning and Mental Well-Being in Hemodialysis Patients in Japan: A Cross-Sectional Design. Health Qual. Life Outcomes.

[24] Chan R., Dear B.F., Titov N., Chow J., Suranyi M. (2016). Examining Internet-Delivered Cognitive Behaviour Therapy for Patients with Chronic Kidney Disease on Haemodialysis: A Feasibility Open Trial. J. Psychosom. Res..

[25] Agudelo-Botero M., Valdez-Ortiz R., Giraldo-Rodríguez L., González-Robledo M.C., Mino-León D., Rosales-Herrera M.F., Cahuana-Hurtado L., Rojas-Russell M.E., Dávila-Cervantes C.A. (2020). Overview of the burden of chronic kidney disease in Mexico: Secondary data analysis based on the Global Burden of Disease Study 2017. B.M.J. Open..

[26] (2018). United States Renal Data System. 2018 USRDS Annual Data Report: Executive Summary.

[27] González-De-Jesús L.N., Sánchez-Román S., Morales-Buenrostro L.E., Ostrosky-Solís F., Alberú J., García-Ramos G., Marino-Vázquez L.A., McClintock S.M. (2011). Assessment of Emotional Distress in Chronic Kidney Disease Patients and Kidney Transplant Recipients. Rev. De Investig. Clin..

[28] Murillo-Zamora E., Macías-De La Torre A.A., Higareda-Almaraz M.A. (2016). Depression Prevalence among End Stage Renal Disease Patients in Maintenance Hemodialysis. Rev. Med. Inst. Mex. Seguro Soc..

[29] Sanchez-Meza F., Torre A., Castillo-Martinez L., Sanchez-Roman S., Morales-Buenrostro L.E. (2021). Evaluation of Cerebral Dysfunction in Patients with Chronic Kidney Disease Using Neuropsychometric and Neurophysiological Tests. Ren. Fail..

[30] Rodriguez Cuellar C.I., García de la Puente S., Hernández Moraria J., Bojórquez Ochoa A., Filler G., Zaltzman Grishevich S. (2019). High Depression Rates among Pediatric Renal Replacement Therapy Patients: A Cross-Sectional Study. Pediatric Transplant..

[31] Jurado S., Villegas M.E.M.E., Méndez L., Rodríguez F., Loperena V., Varela R., Loperana V., Varela R. (1998). La Estandarización Del Inventario de Depresion de Beck Para Los Residentes de La Ciudad de México. Salud Ment..

[32] Robles R., Varela R., Jurado S., Páez F. (2001). Versión Mexicana Del Inventario de Ansiedad de Beck: Propiedades Psicométricas. Rev. Mex. De Psicol..

[33] Katz M.R., Kopek N., Waldron J., Devins G.M., Tomlinson G. (2004). Screening for Depression in Head and Neck Cancer. Psycho-Oncology.

[34] Debnath S., O’Connor J., Hura C., Kasinath B., Lorenzo C. (2017). Quality of Life and Depression Among Mexican Americans on Hemodialysis: A Preliminary Report. Ther. Apher. Dial..

[35] Sohn B., Oh Y., Choi J., Song J., Lim A., Lee J.P., An J.N., Choi H., Hwang J., Jung H.-Y. (2018). Effectiveness of Group Cognitive Behavioral Therapy with Mindfulness in End-Stage Renal Disease Hemodialysis Patients. Kidney Res. Clin. Pract..

[36] Dehesa-López E., Correa-Rotter R., Olvera-Castillo D., González-Parra C., Baizabal-Olarte R., Orozco-Vega R. (2017). Transcultural Adaptation and Validation of the Mexican Version of the Kidney Disease Questionnaire KDQOL-SF36 Version 1.3. Qual. Life Res..

[37] Cukor D., Coplan J., Brown C., Peterson R.A., Kimmel P.L. (2008). Course of Depression and Anxiety Diagnosis in Patients Treated with Hemodialysis: A 16-Month Follow-Up. Clin. J. Am. Soc. Nephrol..

[38] Pascazio L., Nardone I.B., Clarici A., Enzmann G., Grignetti M., Panzetta G.O., Vecchiet C. (2010). Anxiety, Depression and Emotional Profile in Renal Transplant Recipients and Healthy Subjects: A Comparative Study. Transplant. Proc..

[39] Lin X., Jung L., Liu H., Teng S., Zhang W. (2016). Depressive Symptoms and Associated Factors among Renal-Transplant Recipients in China. Int. J. Nurs. Sci..

[40] Müller H.H., Englbrecht M., Wiesener M.S., Titze S., Heller K., Groemer T.W., Schett G., Eckardt K.-U., Kornhuber J., Maler J.M. (2015). Depression, Anxiety, Resilience and Coping Pre and Post Kidney Transplantation—Initial Findings from the Psychiatric Impairments in Kidney Transplantation (PI-KT)-Study. PloS ONE.

[41] Araujo S.M., de Bruin V.M., Daher E.D.F., Almeida G.H., Medeiros C.A., de Bruin P.F.C. (2012). Daher Risk Factors for Depressive Symptoms in a Large Population on Chronic Hemodialysis. Int. Urol. Nephrol..

[42] Balaban Ö.D., Aydin E., Keyvan A., Yazar M.S., Tuna Ö., Özgüven H.D. (2017). Psychiatric Comorbidity, Sexual Dysfunction, and Quality of Life in Patients Undergoing Hemodialysis: A Case-Control Study. Arch. Neuroypsychiatr..

[43] Cameron J.I., Whiteside C., Katz J., Devins G.M. (2000). Differences in Quality of Life across Renal Replacement Therapies: A Meta-Analytic Comparison. Am. J. Kidney Dis. Off. J. Natl. Kidney Found..

[44] Carrillo Algarra A.J., Moreno Rubio F., Milena Buitrago S. (2015). Chronic Kidney Disease and Kidney Transplantation: Experiences and Overcoming of a Medical Student. Index De Enferm..

[45] Tufan F., Yıldız A., Dogan I., Yıldız D., Sevinir Ş. (2015). Urea to Creatinine Ratio: A Forgotten Marker of Poor Nutritional State in Patients Undergoing Hemodialysis Treatment. Aging Male.

[46] Gok Oguz E., Akoglu H., Ulusal Okyay G., Yayar O., Karaveli Gursoy G., Buyukbakkal M., Canbakan B., Ayli M.D. (2016). Serum Apelin Is Associated with Affective Disorders in Peritoneal Dialysis Patients. Ren. Fail..

[47] Griva K., Kang A.W.C., Yu Z.L., Lee V.Y.W., Zarogianis S., Chan M.C., Foo M. (2016). Predicting Technique and Patient Survival over 12 Months in Peritoneal Dialysis: The Role of Anxiety and Depression. Int. Urol. Nephrol..

[48] Yoong R.K., Mooppil N., Khoo E.Y., Newman S.P., Lee V.Y., Kang A.W., Griva K. (2017). Prevalence and Determinants of Anxiety and Depression in End Stage Renal Disease (ESRD). A Comparison between ESRD Patients with and without Coexisting Diabetes Mellitus. J. Psychosom. Res..

[49] Halen N., Cukor D., Constantiner M., Kimmel P.L. (2012). Depression and Mortality in End-Stage Renal Disease. Curr. Psychiatry Rep..

[50] Böell J.E.W., da Silva D.M.G.V., Hegadoren K.M. (2016). Sociodemographic Factors and Health Conditions Associated with the Resilience of People with Chronic Diseases: A Cross Sectional Study. Rev. Lat. -Am. De Enferm..

[51] Dąbrowska-Bender M., Dykowska G., Żuk W., Milewska M., Staniszewska A. (2018). The Impact on Quality of Life of Dialysis Patients with Renal Insufficiency. Patient Prefer. Adherence.

[52] Xu Y., Shao J., Zeng W., Wu X., Huang D., Zeng Y., Wu J. (2021). Depression and Creativity During COVID-19: Psychological Resilience as a Mediator and Deliberate Rumination as a Moderator. Front. Psychol..

[53] Sauvanaud F., Kebir O., Vlasie M., Doste V., Amado I., Krebs M.O. (2017). Bénéfice d’un Programme d’éducation Thérapeutique Agréé Sur La Qualité de Vie et Le Bien-Être Psychologique de Sujets Souffrant de Schizophrénie. Encephale.

[54] Devins G.M., Hollomby D.J., Barré P.E., Mandin H., Taub K., Paul L.C., Guttmann R., Binik Y.M. (2000). Long-Term Knowledge Retention Following Predialysis Psychoeducational Intervention. Nephron.

[55] Saraireh F., Aloush S., Azzam M., Bashtawy M. (2018). The Effectiveness of Cognitive Behavioral Therapy versus Psychoeducation in the Management of Depression among Patients Undergoing Haemodialysis. Issues Ment. Health Nurs..

[56] Tolin D.F., Frost R.O., Steketee G., Muroff J. (2015). Cognitive Behavioral Therapy for Hoarding Disorder: A Meta-Analysis. Depress. Anxiety.

[57] Bernardo N., Cesario Bellantuono A.M.-P. (2012). Eficacia de La Terapia Cognitivo Conductual En La Prevención de La Depresión posparto. Rev Chil Obs. Ginecol.

[58] Gheshlagh R.G., Sayehmiri K., Ebadi A., Dalvandi A., Dalvand S., Tabrizi K.N. (2016). Resilience of Patients With Chronic Physical Diseases: A Systematic Review and Meta-Analysis. Iran Red Crescent Med J. 2016.

[59] Edward K.L. (2005). Resilience: A Protector from Depression. J. Am. Psychiatr. Nurses Assoc..

[60] Shrivastava A., Desousa A. (2016). Resilience: A Psychobiological Construct for Psychiatric Disorders. Indian J. Psychiatr..

[61] Han M.-H., Nestler E.J. (2017). Neural Substrates of Depression and Resilience. Neurotherapeutics.

[62] Won S., Ye G., Rim H.D., Jae S. (2018). Mediating Effect of Resilience on the Association between Emotional Neglect and Depressive Symptoms. Psychiatry Investig..

[63] Evers A.W.M., Zautra A., Thieme K. (2011). Stress and Resilience in Rheumatic Diseases: A Review and Glimpse into the Future. Nat. Rev. Rheumatol..

[64] Crump C., Sundquist J., Winkleby M.A., Sundquist K. (2016). Stress Resilience and Subsequent Risk of Type 2 Diabetes in 1.5 Million Young Men. Diabetologia.

[65] Li X., Harrison S.E., Fairchild A.J., Chi P., Zhao J., Zhao G. (2017). A Randomized Controlled Trial of a Resilience-Based Intervention on Psychosocial Well-Being of Children Affected by HIV/AIDS: Effects at 6- and 12-Month Follow-Up. Soc. Sci. Med..

[66] Ozbay F., Douglas J., Dimoulas E., Morgan C.A., Charney D., Southwick S. (2007). Social Support and Resilience to Stress. Psychiatry.

[67] Beth U. (2016). Promoting Resilience Among Patients with End Stage Renal Disease. Nephrol. Nurs. J..

[68] Rosenberg A.R., Baker K.S., Syrjala K.L., Back A.L., Wolfe J. (2013). Promoting Resilience among Parents and Caregivers of Children with Cancer. J. Palliat. Med..

[69] Cal S., Ribeiro L., Glustak E., Barreto M. (2015). Resilience in Chronic Diseases: A Systematic Review. Cogent Psychol..

[70] Cukor D., Cohen S.D., Peterson R.A., Kimmel P.L. (2007). Psychosocial Aspects of Chronic Disease: ESRD as a Paradigmatic Illness. J. Am. Soc. Nephrol..

