# Supplementary material for: Resilience: A Protective Factor from Depression and Anxiety in Mexican Dialysis Patients
Source: Int J Environ Res Public Health. 2021 Nov 14;18(22):11957. doi: 10.3390/ijerph182211957 (PMC8620979; doi:10.3390/ijerph182211957)
Supplement: Supplementary file 1 [file ijerph-18-11957-s001.zip › SupplementaryMaterial_SectionS1_DetailedRegressionAnalysis.pdf]

Supplementary material

Section S1: Detailed description of the logistic regression analysis

In each variable that had a significant difference between the groups with or without depression symptoms, the association with the presence of symptoms of depression (total depression score ≥ 10 points) or the presence of anxiety (total anxiety score ≥ 7 points) was evaluated by logistic regression analysis, and results are reported as odds ratio (95% confidence interval). The odds ratio of each variable was estimated with a univariate logistic regression model. For instance, the odds ratio of age as an independent variable associated with the presence of depression was estimated with the model of Equation 1. The odds ratio for age was obtained from the estimated parameter β_1, i.e., odds ratio = e^{β_1}. Since age is a continuous variable (measured in years), an odds ratio greater than one is interpreted as the odds of having depression symptoms for every year of age increase in a patient.

$$\text{Presence of depression} = \beta_0 + \beta_1 \cdot \text{age} \quad (1)$$

Equation 2 shows the logistic regression model used to estimate the odds ratio of the presence of depression for a categorical variable, i.e., having an educational level of primary school, as compared to having an education level of secondary school or higher.

$$\text{Presence of depression} = \beta_0 + \beta_1 \cdot \text{primary school} \quad (2)$$

If the odds ratio estimated from Equation 2 is greater than one, it is interpreted that a patient with an educational level of primary school is more likely (as larger odds) of having the presence of depression symptoms than a patient with an educational level of secondary school or higher.

Then, multivariate logistic regression models were used to calculate adjusted odds ratios for each variable. A regression model (Equation 3) included all the sociodemographic variables associated with the presence of depression.

$$\text{Presence of depression} = \beta_0 + \beta_1 \cdot \text{age} + \beta_2 \cdot \text{primary school} + \beta_3 \cdot \text{drug use} + \beta_4 \cdot \text{comorbidities} + \beta_5 \cdot \text{previous transplant} + \beta_6 \cdot \text{waiting transplant} \quad (3)$$

Then, other multivariate logistic regression models (Equations 4 to 7) were used to estimate the adjusted odds ratios for psychological variables and quality of life, by considering all sociodemographic variables in Equation 3 and adding one psychological variable in each model. This allows the estimation of the magnitude of the association between each psychological variable and quality of life with the presence of depression, by adjusting for the sociodemographic variables that could be considered confounding factors.

$$\text{Presence of depression} = \beta_0 + \beta_1 \cdot \text{age} + \beta_2 \cdot \text{primary school} + \beta_3 \cdot \text{drug use} + \beta_4 \cdot \text{comorbidities} + \beta_5 \cdot \text{previous transplant} + \beta_6 \cdot \text{waiting transplant} + \beta_7 \cdot \text{anxiety score} \quad (4)$$

$$\text{Presence of depression} = \beta_0 + \beta_1 \cdot \text{age} + \beta_2 \cdot \text{primary school} + \beta_3 \cdot \text{drug use} + \beta_4 \cdot \text{comorbidities} + \beta_5 \cdot \text{previous transplant} + \beta_6 \cdot \text{waiting transplant} + \beta_7 \cdot \text{resiliency score} \quad (5)$$

$$\text{Presence of depression} = \beta_0 + \beta_1 \cdot \text{age} + \beta_2 \cdot \text{primary school} + \beta_3 \cdot \text{drug use} + \beta_4 \cdot \text{comorbidities} + \beta_5 \cdot \text{previous transplant} + \beta_6 \cdot \text{waiting transplant} + \beta_7 \cdot \text{cognitive distortions score} \quad (6)$$

$$\text{Presence of depression} = \beta_0 + \beta_1 \cdot \text{age} + \beta_2 \cdot \text{primary school} + \beta_3 \cdot \text{drug use} + \beta_4 \cdot \text{comorbidities} + \beta_5 \cdot \text{previous transplant} + \beta_6 \cdot \text{waiting transplant} + \beta_7 \cdot \text{quality of life score} \quad (7)$$

A regression model (Equation 8) included all the sociodemographic and clinical variables associated with the presence of anxiety since all of them had significant odds ratios in the univariate logistic regression analysis.

$$\text{Presence of anxiety} = \beta_0 + \beta_1 \cdot \text{albumin} + \beta_2 \cdot \text{working status} + \beta_3 \cdot \text{previous transplant} \quad (8)$$

Then, other regression models (Equations 9 to 12) were used to estimate the adjusted odds ratios for psychological variables and quality of life, by considering all sociodemographic variables in Equation 8 and adding one psychological variable in each model. This allows the estimation of the magnitude of the association between each psychological variable and quality of life with the presence of anxiety, by adjusting for the sociodemographic variables that could be considered confounding factors.

$$\text{Presence of anxiety} = \beta_0 + \beta_1 \cdot \text{albumin} + \beta_2 \cdot \text{working status} + \beta_3 \cdot \text{previous transplant} + \beta_4 \cdot \text{depression score} \quad (9)$$

$$\text{Presence of anxiety} = \beta_0 + \beta_1 \cdot \text{albumin} + \beta_2 \cdot \text{working status} + \beta_3 \cdot \text{previous transplant} + \beta_4 \cdot \text{resiliency score} \quad (10)$$

$$\text{Presence of anxiety} = \beta_0 + \beta_1 \cdot \text{albumin} + \beta_2 \cdot \text{working status} + \beta_3 \cdot \text{previous transplant} + \beta_4 \cdot \text{cognitive distortions score} \quad (11)$$

$$\text{Presence of anxiety} = \beta_0 + \beta_1 \cdot \text{albumin} + \beta_2 \cdot \text{working status} + \beta_3 \cdot \text{previous transplant} + \beta_4 \cdot \text{quality of life score} \quad (12)$$
