# Supplementary material for: Resilience: A Protective Factor from Depression and Anxiety in Mexican Dialysis Patients
Source: Int J Environ Res Public Health. 2021 Nov 14;18(22):11957. doi: 10.3390/ijerph182211957 (PMC8620979; doi:10.3390/ijerph182211957)
Supplement: Supplementary file 1 [file ijerph-18-11957-s001.zip › SupplementaryMaterial_TableS1.pdf]

Supplementary material

Table S1. Relevant previous research regarding the factors associated with depression and anxiety, distorted thoughts, and psychological resilience in ESRD patients treated with hemodialysis.

Ref.*	Studied population	Depression	Anxiety	Resilience	Distorted thoughts
5	N = 88 (31% female)<br>Country: Pakistan<br>RRT: HD	Prevalence = 76%<br>Related factors:<br>female sex and social support<br>Not related:<br>Age, duration of dialysis, marital status, education	Not included	Not included	Not included
4	N= 359 (42% female)<br>Country: USA<br>RRT: HD, DP	Prevalence = 51 %<br>Related factors:<br>Gender, age and cardiovascular disease.<br>Not related:<br>Diabetes type 2, potassium.	Not included	Not included	Not included
15	N= 208 (48% female)<br>Country: USA<br>RRT: HD	Prevalence : 57%<br>Related factors:<br>Not related:<br>Non organizational religiosity	Not included	Related factors:<br>Higher medication adherence and high perception to quality of life.<br>Not related:<br>organizational religiosity.	Not included
9	N=255 (53% female)<br>Country: Mexico<br>RRT: HD, DP	Related factors:<br>Catastrophism, cognitive distortions	Related factors:<br>Depression, cognitive distortions	Not included	Related factors:<br>Depression, anxiety
16	N=196 (62% female)<br>Country: China<br>RRT: HD, DP	Not included	Not included	Related factors:<br>Educational level, employment status, dialysis duration.<br>Not related:<br>Intrusive rumination, deliberate rumination	Not included
18	N= 56 (35.7% female)<br>Country: USA<br>RRT: Predialysis	Prevalence: 65%<br>Related Factors:<br>Anxiety, low level to quality of life perception.<br>Not related:<br>Resilience	Prevalence: 22%<br>Related Factors: :<br>Separation anxiety<br>Not related:<br>Quality of life	Related factors:<br>Education level<br>Not related:<br>Gender	Not included
	N= 252 (37.7% female)	Prevalence 52%<br>Related factors:	Related factors:	Related factors:<br>Age, partnership,	Not included

	anxiety, partnership	Depression	hemodialysis
Country: Germany	Not related:	Not related:	duration.
	Age, hemodialysis	Gender, age,	Not related:
RRT: HD, DP, RT,	duration, gender.	hemodialysis	Gender.
		duration.	

* Details of each reference number is enlisted on the References section of the manuscript “González-Flores, C.J.; García-García, G.; Lerma, A.; Perez-Grovas, H.; Meda-Lara, R.M.; Guzmán-Saldaña, R.M.E., Lerma, C. Resilience: A protective factor from depression and anxiety in Mexican dialysis patients. Int. J. Environ. Res. Public Health 2021.
